# Pediatric asthma and altitude: a complex interplay between different environmental factors

**DOI:** 10.1186/s13052-023-01492-x

**Published:** 2024-03-06

**Authors:** Laura Bisoffi, Giovanni Sassudelli, Fabio Agostinis, Annalisa Cogo, Renato Cutrera, Irene Dalpiaz, Maria Elisa Di Cicco, Battista Guidi, Stefania La Grutta, Andrea Miceli, Francesca Mori, Giorgio Piacentini, Diego Peroni, Deborah Snjiders, Mattia Giovannini, Ermanno Baldo

**Affiliations:** 1 Department of Health Sciences, University of Florence, Florence, Italy; 2 Pediatric Outpatient Service, Bergamo, Italy; 3 Center for Exercise and Sport Science, University of Ferrara, Ferrara, Italy; 4 Institute Pio XII, Misurina, Italy; 5 Pediatric Pulmonology Unit, Academic Department of Pediatrics, Bambino Gesù Children’s Hospital, IRCCS, Rome, Italy; 6 Department of Clinical and Experimental Medicine, Section of Pediatrics, University of Pisa, Pisa, Italy; 7 Hospital and Territorial Pediatrics Unit, Pavullo Hospital, Pavullo nel Frignano, Italy; 8 National Research Council, Institute of Translational Pharmacology (IFT), Palermo, Italy; 9 Allergy Unit, Meyer Children’s Hospital IRCCS, Florence, Italy; 10 Pediatric Clinic, Università degli Studi di Verona, Verona, Italy; 11 Department of Woman and Child Health (SDB), University of Padova, Padua, Italy; 12 “Giovan Battista Mattei” Research Institute, Stenico, Italy

**Keywords:** Asthma, Altitude, Environment, Mountain, Pediatrics

## Abstract

Asthma is one of the most common non-communicable diseases, and its prevalence and morbidity are influenced by a wide array of factors that are only partially understood. In addition to individual predisposition linked to genetic background and early life infections, environmental factors are crucial in determining the impact of asthma both on an individual patient and on a population level.

Several studies have examined the role of the environment where asthmatic subjects live in the pathogenesis of asthma. This review aims to investigate the differences in the prevalence and characteristics of asthma between the pediatric population residing at higher altitudes and children living at lower altitudes, trying to define factors that potentially determine such differences. For this purpose, we reviewed articles from the literature concerning observational studies assessing the prevalence of pediatric asthma in these populations and its characteristics, such as spirometric and laboratory parameters and associated sensitization to aeroallergens.

Despite the heterogeneity of the environments examined, the hypothesis of a beneficial effect of residing at a higher altitude on the prevalence of pediatric asthma could be confirmed, as well as a good profile on airway inflammation in asthmatic children. However, the possibility of a higher hospitalization risk for asthma in children living at higher altitudes was demonstrated. Moreover, a positive association between residing at a higher altitude and sensitization to pollens and between lower altitude and sensitization to house dust mites could be confirmed in some pediatric patients, even if the results are not homogeneous, probably due to the different geographical and climatic regions considered. Nonetheless, further studies, e.g., extensive and international works, need to be conducted to better understand the complex interplay between different environmental factors, such as altitude, and the pathogenesis of asthma and how its prevalence and characteristics could vary due to climate change.

## Introduction

Asthma is one of the most common non-communicable diseases in the pediatric population, characterized by high morbidity, elevated socio-economic costs, and non-negligible mortality [1]. The prevalence of asthma clinical manifestations in school-aged children has been changing worldwide in the last decades, showing relative stability in upper-middle-income and high-income countries (i.e., Western Europe and North America) and, on the other hand, a marked increase in low-income and middle-income ones [2]. The prevalence of asthma differs among countries and even among different regions within the same country depending on various and strictly interconnected pathogenic factors, characterizing a multi-factorial model with complex relationships between genes and environment [3–5].

Some of these factors directly depend on the subject’s characteristics, including genetic background, family history of asthma or other atopic diseases, airway microbiome and viral infections [6, 7]. Other factors are more generally connected to the subject’s living environment. The latter includes rural or urban setting, altitude, climatic conditions (e.g., temperature and humidity), exposure to airway irritant factors (e.g., air pollution [8–10] and tobacco smoke) and to sensitizing aeroallergens (e.g., house dust mites and pollens).

Several studies have been published in the last decades concerning the impact of altitude on pediatric asthma. Specifically, research focused on the prevalence of asthma among children permanently residing at altitude and on the effect of sojourns at altitude as a therapy for asthmatic children usually residing at a lower altitude (i.e., “climate-therapy” or “alpine-therapy” [11–14]). The European Academy of Allergy & Clinical Immunology published a position paper which examined the currently available literature concerning alpine altitude climate treatment. It highlighted its possible role as an adjunct therapy for patients with poorly controlled asthma, given its beneficial effects on various clinical and lung function parameters [15]. Based on its effects on human physiology in healthy individuals, altitude can be classified into low (< 1200 m), moderate (1200–2500 m), high (2500–3500 m), very high (3500–5800 m), and extreme (> 5800 m) [16].

Specific features of altitude environment have been hypothesized to be associated with the effect of altitude on both the prevalence of asthma in the residing population and its effect as a therapy on asthmatic children. Mountainous areas are generally thinly populated, with low levels of vehicular traffic and low density of industrial activities, with consequent minimal air pollution. Moreover, certain climate conditions (e.g., low medium annual temperatures and reduced humidity levels) are associated with lower indoor levels of house dust mites (HDM) [17]. Furthermore, reduced humidity determines a lower level of mold in homes at high altitude. Additionally, lower atmospheric pressure reduces air viscosity, thus generating lower resistance in the airways and favouring better pulmonary flows and volumes [3]. Finally, UV irradiation is increased at high altitudes, with consequently more significant vitamin D production and a possible immune-modulating effect [18].

Notably, high-altitude climates differ depending on latitude. For example, the mountain climate in the South American tropical belt is not comparable with the alpine climate, the first being hotter and more humid than the second. Moreover, differences between pollen-derived triggers in these environments could influence the prevalence of pediatric asthma in the populations described [19].

Moreover, since the relation between asthma and altitude is tightly connected to climate, planetary climate change cannot be ignored with various effects on the incidence of pediatric asthma through both direct and indirect mechanisms. Climate change is associated with several asthma risk factors, such as levels of air pollution in urban areas, variations in the geographic distribution of some plants, and earlier onset and elongation of the pollen season, determining an increased pollen production. Moreover, rising humidity has an essential impact on the presence of allergens, such as molds [19–22].

This review aims to investigate the differences in the prevalence and characteristics of asthma in the pediatric population residing at higher altitudes compared to children living at a lower altitude, trying to identify the factors that could determine such differences. For this purpose, we reviewed articles from the literature concerning observational studies assessing the prevalence of pediatric asthma in these populations and its characteristics, such as spirometric and laboratory parameters and associated sensitization to aeroallergens.

## Methods

A literature search in Medline through Pubmed was independently performed by two researchers (LB and GS) using the following strategy (asthma OR asthm* OR bronchoresponsiveness OR wheezing OR bronchial responsiveness OR FEV*) AND (children OR child OR pediatric OR pediatr*) AND (altitude OR climate therapy OR climatotherapy OR mountain).

**Fig. 1:**
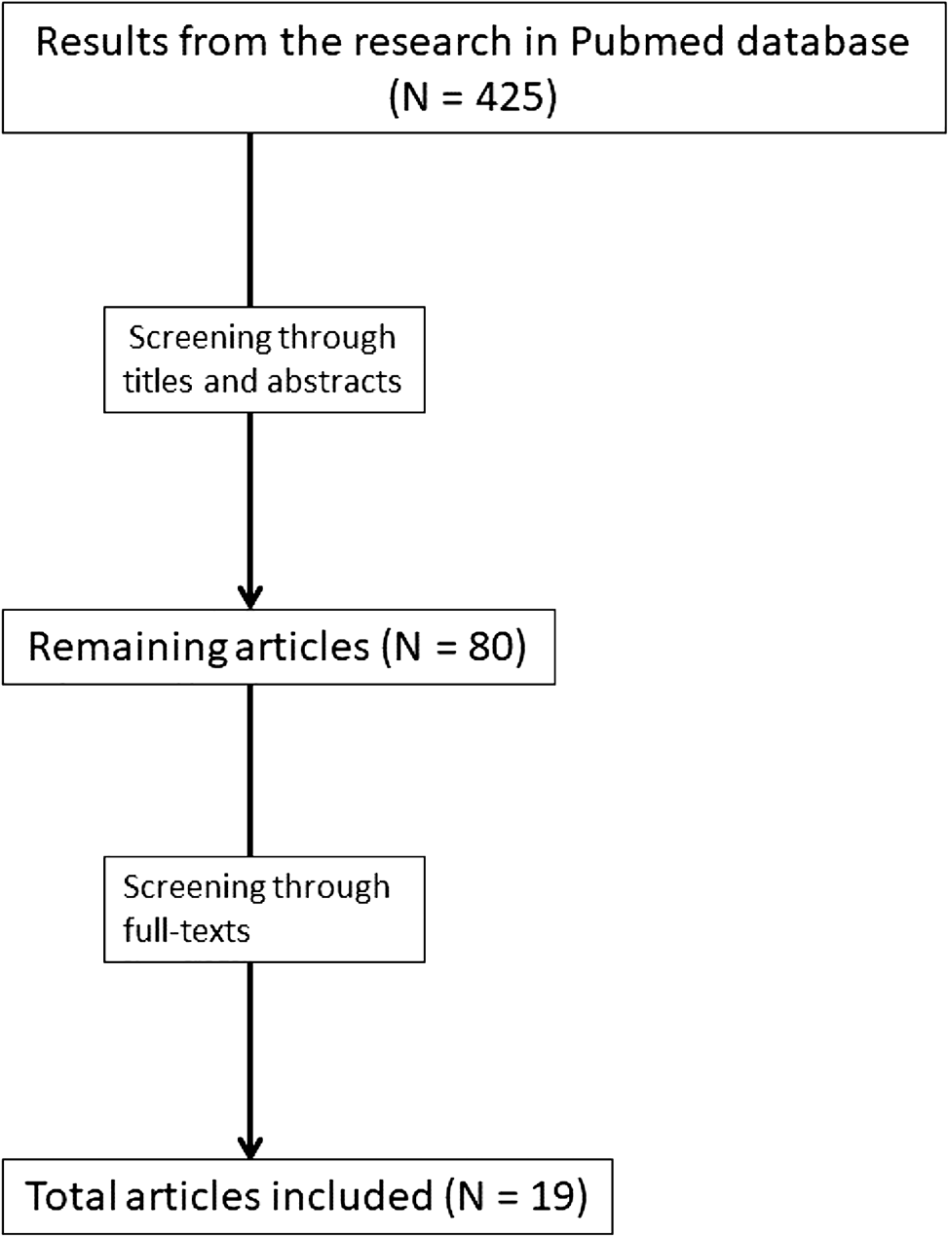
The result of the research and the following article selection

This research referenced 425 articles, published up to January 2022. After analyzing many titles and abstracts, 345 results were excluded because they were written in languages other than English, did not concern the aim of the review, did not investigate pediatric populations, or they were commentaries, letters, repeated published articles and case reports. The full texts of the 80 remaining studies were subsequently evaluated by all of the authors, and 61 were excluded for the following reasons: (1) they concerned arguments that did not pertain to the aim of the review, (2) they analyzed populations of both children and adults but could not extrapolate isolated data about the pediatric group, (3) they considered “living at high altitude” a population actually residing at an altitude lower than 900 m, and (4) regarding clinical trials, they used mountain stay as a therapy for asthma. Therefore, 19 results concerning pediatric observational studies that assess the prevalence of asthma related to altitude and its characteristics were included. In both screening phases, disagreements were resolved through negotiation. The result of the research and the following article selection is shown in Fig. 1.

Meanwhile, the following data were extracted from the included articles: study population, environments examined, study outcomes, and main results. We categorized the selected articles according to the type of outcome as follows:

1. Studies investigating the prevalence of asthma assessing respiratory clinical manifestations and previous clinical diagnoses of asthma;
2. Studies including instrumental objective parameters concerning asthma;
3. Studies evaluating the risk of hospitalization for asthma;
4. Studies assessing the prevalence of sensitization to specific aeroallergens.

The extracted information was recorded in tabular format, further subdividing the arcticles among the aforementioned four categories **(**Tables 1, 2, 3 and 4**)**, and the results were summarized.

**Table 1:** Summary of studies investigating the prevalence of asthma assessing respiratory clinical manifestations and previous clinical diagnoses of asthma

Reference (year of publication)	Study population (n)	Environments examined	Main study outcomes
Gourgoulianis et al. (2001) [31]	874	Greece, three groups living between 0 and 500 m, 501 and 800 m and between 801 and 1,200 m above sea level	Questionnaires evaluating asthma prevalence, asthma-related morbidity in terms of days of school lost and nocturnal dyspnea
Soto-Quiros et al. (2002) [27]	9,931	Costa Rica, coastal regions (below 1,000 m) and highland regions (above 1,000 m)	Questionnaires evaluating asthma prevalence and clinical manifestations
Kawada et al. (2004) [35]	17,402	Japan, three subgroups living between 0-100 m, 100–200 m and 200-1,200 m	Questionnaire to evaluate asthma prevalence
Weiland et al. (2004) [25]	Unspecified number of children between 6 and 7 years old, 463,801 subjects between 13 and 14 years old	Worldwide, in a variety of environments at different altitudes	Questionnaire evaluating the prevalence of asthma clinical manifestations
Yangzong et al. (2006) [29]	2,026	Tibet, 4,300-4,400 m	Questionnaire to evaluate asthma prevalence and clinical manifestations
Droma et al. (2007) [30]	3,190	Tibet, 3,659 m	Questionnaire to evaluate asthma prevalence and clinical manifestations
Kurt et al. (2007) [32]	25,843	Turkey, 14 cities at different altitudes	Questionnaire evaluating asthma clinical manifestations
Mallol et al. (2010) [28]	165,917	Multiple countries in South America, multiple cities at different altitudes and environments	Questionnaire evaluating asthma prevalence and clinical manifestations
Del-Rio-Navarro et al. (2020) [34]	77,179	Mexico, 14 cities at different altitudes	Questionnaire to evaluate asthma prevalence

**Table 2:** Summary of studies including instrumental objective parameters concerning asthma

Reference (year of publication)	Study population (n)	Environments examined	Main study outcomes
Sporik et al. (1995) [36]	525 in Phase 1, 120 in Phase 2	USA, a city at 2,195 m of altitude	Questionnaire assessing asthma prevalence, asthma therapy, ER visits and hospital admission due to asthma, SPT for aeroallergens, serum total IgE dosage, baseline spirometry, bronchial hyperresponsiveness testing and bronchodilator test
Giroux et al. (2001) [37]	126	France, a city at 141 m and a specialized residential centre in a mountain area at 1000 m	FeNO
Giroux et al. (2002) [40]	140	France, a city at 141 m and a specialized residential centre in a mountain area at 1000 m	FeNO, expired NH3, urinary NH4+, sodium, potassium and urea
Ramirez et al. (2021) [41]	93	Bogota, Colombia (2640 m)	Several IOS and spirometric parameters at baseline and after bronchodilator

**Table 3:** Summary of studies evaluating risks of hospitalization from asthma

Reference (year of publication)	Study population (n)	Environments examined	Main study outcomes
Kiechl-Kohlendorfer et al. (2007) [44]	33,808 live-born subjects	Austria, a mountainous region divided in subgroups according to altitude (< 900 m, 900-1,199 m, > 1,200 m)	Prospective evaluation of the risk of hospitalization for atopic asthma

**Table 4:** Summary of studies assessing the prevalence of sensitization to specific aeroallergens

Reference (year of publication)	Study population (n)	Environments examined	Main study outcomes
Charpin et al. (1991) [45]	933	France, a coastal town and a town at 1,326 m in the Alps	Prevalence of ENT and respiratory symptoms, positive SPT to pollens or HDM
Ozkaya et al. (2015) [46]	1,121	Turkey, a major city on the coast and another city at 1,800-2,000 m	Questionnaire evaluating asthma prevalence, SPT, total IgE
Duenas-Meza et al. (2018) [47]	61, all with severe asthma	Colombia, all living at high altitude (2,500-3,500 m)	Questionnaire assessing asthma clinical manifestations and control, baseline and post-bronchodilator spirometry, SPT, total serum IgE, FeNO
Abiad et al. (2020) [49]	919, all asthmatic	Lebanon, the population was subdivided between those living below or above 900 m	SPT for aeroallergens to evaluate atopy in asthmatic patients
Ochoa-Avilés et al. (2020) [50]	353	Ecuador, a city in the Andes at 2,550 m	Questionnaire assessing asthma prevalence and clinical manifestations, SPT for aeroallergens

## Results

### Studies investigating the prevalence of asthma assessing respiratory clinical manifestations and previous clinical diagnosis of asthma

The International Study of Asthma and Allergies in Childhood (ISAAC) is a randomized cross-sectional multicenter study composed of three phases [23, 24]. ISAAC Phase One assessed worldwide the prevalence and severity of several diseases, including asthma, through a questionnaire in a cohort of adolescents aged 13–14 years old and in another cohort of children aged 6–7 years old. ISAAC Phase Two involved a group of 10–12-year-olds in specific populations characterized by a different prevalence of the diseases found in Phase One and different characteristics, such as environmental exposures, management, or genetic factors. This phase included a more detailed questionnaire and some objective data, such as clinical evaluation, spirometry, bronchial hyperresponsiveness testing, skin prick testing, and serum IgE dosage. ISAAC Phase Three was conducted in the same countries in which ISAAC Phase One took place as well as some others, using the same study design and questionnaire as in Phase One, in order to assess changes in the prevalence of the pathologies investigated over time. Many articles have been published regarding data collected from the ISAAC study, both worldwide and in specific countries or regions. We reviewed all those assessing the prevalence of asthma and related pathologies at high altitudes or comparing populations who reside at different altitudes.

Weiland et al. [25] analyzed data from ISAAC Phase One, assessing the prevalence of asthma signs and symptoms in both 6–7- and 13–14-year-old patients living in different environmental and climatic conditions worldwide. Data were adjusted for different countries’ Gross National Product (GNP), as it is known to be positively associated with the prevalence of atopic diseases [26]. Within the various environmental conditions analyzed, altitude showed a negative association with the prevalence of asthma clinical manifestations assessed by a written questionnaire in Western Europe countries: an increase in altitude of 100 m was associated with a decrease in the prevalence of wheezing in the previous 12 months for the 13–14-year-old group and the 6–7-year-old group. For both age groups worldwide, a similar trend was demonstrated, but the 95% CI did not exclude zero. The prevalence of asthma clinical manifestations assessed by video questionnaire (concerning the 13–14-year-old group only) also showed a similar trend but with the 95% CI including zero both in Western Europe and worldwide.

Soto-Quiros et al. [27] investigated the prevalence of asthma signs and symptoms in Costa Rica, using data collected within ISAAC Phase One in both age groups. Living environments were classified as coastal regions (below 1,000 m) and highland regions (above 1,000 m). When the two age groups were considered as a whole, the prevalence of a history of wheezing, wheezing during the last 12 months, and clinical diagnosis of asthma was not significantly different between patients living in the two environments. On the other side, living in the coastal regions was significantly associated with a higher prevalence of dry cough in the last 12 months and wheezing after exercise. Moreover, only in the 13–14 age group, a significant positive association was found between the history of wheezing and living at an altitude above 1000 m.

Mallol et al. [28] assessed the prevalence of asthma clinical manifestations in Latin America, analyzing data collected during ISAAC Phase Three in adolescents between 13 and 14 years old. When altitude was considered as a continuous variable, a significant inverse correlation was found between altitude and lifetime asthma (r = -0.43), current wheezing (r = -0.27), and frequent sleep disturbance (r = -0.44). However, once the eight centers located above 2000 m were removed from the correlation model, the association between the prevalence of asthma signs and symptoms and altitude was not significant anymore.

Two different studies, published by Yangzong et al. [29] and Droma et al. [30], investigated the prevalence of asthma in regions located at high altitudes in Tibet (4300–4400 m in the first study and 3659 m in the second), involving adolescents 12–14 and 13–14 years of age, respectively. Both studies did not have a control population at lower altitudes. However, data were collected in Tibet within ISAAC Phase Three and were therefore comparable with those referring to other countries. In particular, both studies showed a prevalence of asthma-related signs and symptoms among the lowest ever reported worldwide. For example, according to data analyzed by Droma et al. [30], the reported prevalence was 1.4% for having ever experienced wheezing, 0.8% for current wheezing, and 1.1% for lifetime clinical diagnosis of asthma. Furthermore, the majority of subjects in Tibet reported mild clinical manifestations, not seriously threatening their daily activities.

Another study assessing the prevalence and morbidity of asthma through the submission of questionnaires was conducted in Thessaly in Central Greece; data were analyzed and published by Gourgoulianis et al. [31]. Children aged 6–12 years old were enrolled and categorized according to the altitudes of their living environments: between 0 and 500 m, between 501 and 800 m, and between 801 and 1200 m above sea level. The prevalence of asthma among subjects living in the mountains (801–1200 m) was significantly lower than that of children living between sea level and 500 m (7.2% versus 15.8%, respectively); children living at 501–800 m had an intermediate prevalence of asthma (10%). Moreover, asthma in children living in the mountains showed lower morbidity, quantified in days of absence from school per year and nights with dyspnea per year.

In 2004, a multicenter cross-sectional questionnaire-based study named PARFAIT (Prevalence and Risk Factors of Allergies in Turkey) was performed, assessing the prevalence of allergic diseases, including asthma, in the pediatric population. Collected data were published by Kurt et al. [32] and included subjects aged 6–17 years old from several cities all around Turkey; all the enrolled subjects completed a questionnaire about their signs and symptoms in the previous 12 months. After adjustment for age, sex, and rural or urban living environment, both in univariate analysis and in multivariate logistic regression, living at an altitude below 1000 m was significantly associated with a higher prevalence of all allergic diseases. In particular, the prevalence of asthma and wheezing in the last 12 months showed an OR (Odds Ratio) of 1.45 and 1.58, respectively, with 95% confidence intervals both excluding zero, confirming the trend shown by the studies mentioned above. Moreover, the prevalence of asthma and wheezing was associated with higher mean yearly temperature and higher mean yearly outdoor humidity, typical climate conditions of coastal regions in Turkey.

Since 2012, a cross-sectional multicenter questionnaire-based study named GAN (Global Asthma Network) has been collecting data about the prevalence of asthma worldwide, in particular in middle-income and low-income countries [33]. The GAN study involved two age groups (6–7 and 13–14 years old) using the same ISAAC questionnaire in order to compare data. In particular, Del-Rio-Navarro et al. [34] analyzed and published data collected in Mexico within the GAN study in the period 2016–2019. Subjects of both age groups were enrolled in several cities in Mexico, and data were analyzed for the prevalence of wheezing and asthma and for associated factors, including the altitude of the cities (from 7 to 2667 m above sea level). Specifically, the authors found a negative association between altitude and the prevalence of asthma clinical manifestations for both age groups, confirming living at a high altitude, in particular above 1500 m, as a factor against asthma.

Unlike most of the studies mentioned so far, not all studies in our research support a negative association between altitude and the prevalence of asthma.

Indeed, Kawada et al. [35] published a cross-sectional questionnaire-based study conducted in the Gunma Prefecture in Japan and involving children aged 0–4 years old whose parents completed the ATS-DLD (American Thoracic Society – Division of Lung Diseases) questionnaire on respiratory clinical manifestations; children were considered asthmatic if an affirmative answer was given to all six items of the questionnaire. Subjects’ living environment was classified according to altitude: 0–100 m, 100–200 m, and 200–1200 m. In univariate analysis, the prevalence of asthma among boys living at 200–1200 m was significantly higher than among those living at a lower altitude. On the contrary, it did not significantly differ between girls living at different altitudes. In multiple logistic regression analyses, living above 200 m appeared to be a risk factor for asthma, with an OR of 1.38 (95% CI 1.02–1.87). However, the authors hypothesized that higher levels of air pollution (i.e., from acidic fog around Mt. Akagi), even at this low altitude of the studied region, could partially explain their findings.

Finally, most of the studies included in the present section supported the hypothesis of a beneficial effect of living at high altitudes on the prevalence of pediatric asthma. In particular, a critical weight must be given to data from the ISAAC study, thanks to the enormous number of patients and countries involved worldwide and to the use of a standardized questionnaire over time.

Various hypotheses have been formulated to explain the effect of high altitude on the prevalence of pediatric asthma. Most of the authors agree about lower levels of air pollution, house dust mites (HDM) exposure, and specific climatic characteristics, such as lower mean temperature and lower outdoor humidity. These characteristics mainly correspond to the high altitude in Western European countries, where the negative association between asthma and altitude appears, indeed, to be stronger, according to the ISAAC Study.

A major limitation to this kind of study is the fact that the prevalence of asthma is assessed only by questionnaires on respiratory signs and symptoms, and clinical diagnoses of wheezing or asthma are directly performed during data collection, in the absence of any objective evaluation.

### Studies including instrumental objective parameters concerning asthma

Sporik et al. [36] analyzed data collected within a cross-sectional study performed in Los Alamos (New Mexico, United States of America), a city located at 2195 m of altitude, with an almost mite-free environment and very low levels of air pollution. The study involved adolescents aged 12–14 years old and was organized in two phases. In the first, more than 500 subjects and their parents completed a questionnaire assessing respiratory clinical manifestations, lifetime clinical diagnosis of asthma, use of asthma medications, and emergency room attendances or hospital admissions due to asthma attacks. According to the answers given, they were divided into two groups: those who had had respiratory clinical manifestations and/or treatment for asthma and healthy controls. An equal number of children were randomized in the two groups and invited to attend the second phase of the study. Baseline spirometry was performed. Two different tests were then used to identify subjects with bronchial hyperresponsiveness: those showing baseline FEV-1 (forced expiratory volume in 1 s) below 80% of the predicted volume underwent a bronchodilator test with inhaled albuterol, whereas those with a normal baseline FEV-1 (> 80% of the predicted volume) underwent a bronchoprovocation test with inhaled histamine. Globally, a relevant prevalence of respiratory signs and symptoms was found, with 13% of subjects having had at least one episode of asthma in the previous year, 14% currently taking medications for asthma, and 18% reporting a clinical diagnosis of asthma in their lifetime. However, after analyzing disease severity according to emergency attendances and hospital admissions, only few patients were classified as suffering from severe asthma. These data were then confirmed during the second phase, assessing the level of bronchial hyperresponsiveness. A significant limitation of this study was the absence of a control population living at a lower altitude. Nonetheless, the questionnaire-based prevalence of asthma was compared among children who were born and raised in Los Alamos and those who were not, without finding any significant difference. According to baseline spirometry and bronchial hyperresponsiveness testing performed during the second phase of the study, the prevalence of asthma among the whole population was estimated to be 6.3%, much lower than the questionnaire-based estimated prevalence. It is important to highlight the substantially higher percentage of people reporting a clinical diagnosis of asthma in their lifetime compared with the prevalence of asthma assessed by spirometry and bronchial hyperresponsiveness.

Giroux et al. [37] used another objective parameter, FENO (Fractional Exhaled NO), to compare asthmatic and healthy children living at different altitudes in France. FENO is, in fact, known to be a good proxy indicator of airway inflammation and, potentially, of treatment response in asthmatic patients [38, 39]. This study analyzed FENO levels in three groups of children globally, aged 6–14 years. The first two groups were composed of asthmatic children, residing respectively in Toulouse, a city located in Southern France at an altitude of 141 m, and in a specialized institution for asthmatic children in the Pyrenees, at an altitude of 1000 m. The third group was composed of healthy children living in Toulouse. FENO levels of asthmatic children living at high altitude were significantly lower than those of asthmatic children living in Toulouse and were comparable to those of healthy controls living in Toulouse. Moreover, FENO levels of asthmatic children living in Toulouse showed a significant positive association with levels of atmospheric pollution (atmospheric NO and CO) recorded on previous days. Interestingly, among children living in Toulouse, FENO levels were significantly lower in those treated with inhalatory glucocorticoids than in the untreated ones; this difference was absent among children living at high altitude.

An extension of this study with the same design was then performed to investigate the utility of exhaled levels of NH3 and urinary levels of NH4+, NO3-, urea, and electrolytes as indicators of airway inflammation and ICS response in asthmatic patients; results were published by Giroux et al. [40]. Exhaled levels of NH3 and urinary levels of NH4 + did not differ between asthmatic and healthy children living in Toulouse; on the contrary, they were significantly lower among asthmatic children living in the specialized institution in the mountains. Moreover, no association was demonstrated for either of them, neither with FENO levels nor with ICS therapy. Therefore, the authors concluded that exhaled NH3 and urinary NH4 + are not good indicators of asthma and airway inflammation.

Ramirez et al. [41] employed both spirometry and impulse oscillometry (IOS) to assess baseline lung function parameters and bronchodilator response in a cohort of pediatric patients with stable asthma living in Bogota, Colombia (at 2640 m above sea level). This study showed poor concordance between the two techniques, with a significantly higher proportion of patients showing a positive bronchodilator response when using spirometric parameters than with IOS. The main explanation for this result, according to the authors, is that the decrease in air density at higher altitudes results in a higher maximal flow in large airways with no measurable effect on small-airway resistance. Hence, in their view, this could have influenced spirometric bronchodilator response more than IOS-measured bronchodilator response since the latter measures mainly the resistance of distal airways. However, the superiority of spirometry in bronchodilator response detection when compared to IOS in high-altitude environments contrasts with the findings of some previous studies [42, 43]. However, in accordance with previous findings, in this cohort, age and baseline lung function were significantly related to bronchodilator response when assessed with both techniques.

Finally, according to the studies discussed in this section, a good profile on airway inflammation could be confirmed in asthmatic children. This association is probably related to different factors coexisting at high altitudes, such as lower temperatures, humidity, and exposure to air pollution and allergens.

### Studies evaluating the risk of hospitalization for asthma

Only one study., which was published by Kiechl-Kohlendorfer et al. [44], has been included in this section. It is a prospective birth-cohort study including all live-born infants between 1994 and 1999 in Tyrol, a mountainous region located in the Austrian Alps, with a wide range of permanent residence altitudes (450–1800 m). Within the first 4–6 weeks of life of each subject, data were collected about pregnancy, birth, perinatal course, and demographic characteristics, including the altitude of the place of residence (classified as < 900 m, 900–1199 m, and > 1200 m) and living environment (urban, suburban, or rural). Altitude appeared to be strongly inversely associated with the urban living environment, with the only large city in the region being located at low altitude. Between 2000 and 2005, new data were collected about children from the same cohort who were hospitalized for atopic asthma at the age of 6–10 years old. Therefore, subjects were only included if they had a diagnosis of asthma (at least three episodes of wheezing confirmed by clinical examination on hospital admission) and proof of atopy (positive skin prick testing for at least one common aeroallergen grass pollen mix, hazel, birch pollen, cat dander, house dust mite *Dermatophagoides pteronyssinus*, *Alternaria alternata* and *Cladosporium herbarum* and/or elevation of specific serum IgE). In univariate analysis, the altitude did not show any significant association with hospitalization for atopic asthma. However, in multivariate analysis and after adjusting for living environment, a significant positive association was demonstrated between living at a higher altitude and risk of hospitalization for atopic asthma, with a relative risk (RR) of 1.49 (95% CI 1.05–2.11) for living between 900 and 1200 m and of 2.08 (95% CI 1.45–2.98) for living above 1200 m, both compared to living below 900 m. This finding was confirmed when hospital admissions in different seasons were independently analyzed. Moreover, when the altitude of residence was considered as a continuous variable, an increase in altitude of 100 m was associated with an increase of 7% of the risk of hospitalization for atopic asthma. This study does support the hypothesis that altitude plays a role in asthma by showing a higher risk of hospitalization for atopic asthma in children living at higher altitudes. According to the authors, the main explanation for this finding is the fact that the climate at high altitudes is characterized by low outdoor temperature and air humidity, both of which could act as trigger factors for severe asthma attacks. However, a potential bias could be represented by the fact that people residing at higher altitudes and away from urbanized centers and hospital facilities could have incurred more difficulties in obtaining timely treatment, which could have lead to increases in disease severity and in the number of hospitalizations.

### Studies assessing the prevalence of sensitization to specific aeroallergens

Charpin et al. [45] compared children living in two cities in France: Martigues, on the Mediterranean coast, and Briancon, located in the Alps at 1,326 m. Those living in Briancon were then categorized as natives (born and raised in Briancon) and non-natives. All subjects aged 9–11 years old living in the two cities completed a questionnaire assessing clinical manifestations of asthma; a subsample of children from each city also underwent skin prick testing for common aeroallergens (HDM *Dermatophagoides pteronyssinus*; cat dander; grass pollen). The prevalence of ENT (Ear-Nose-Throat) and respiratory signs and symptoms did not significantly differ between children living in Briancon and in Martigues. However, when natives from Briancon were considered separately, the prevalence of respiratory clinical manifestations appeared to be lower than in the two other groups, but without any statistical significance. Moreover, the two cities differed neither in the prevalence of non-atopic asthma (2% in Briancon and 2.2% in Martigues) nor in that of atopy (defined as a positive skin test to at least one aeroallergen), which was found to be 25.6% in Briancon and 25.1% in Martigues. Nonetheless, children living in the mountains showed a lower proportion of positive skin testing for HDM and a higher proportion of sensitization to grass pollens. This trend appeared to be more evident for natives than for non-natives from Briancon. Consistently with this observation, natives from Briancon showed a significantly lower prevalence of asthma with positive prick testing to HDM than both non-natives from Briancon and children living in Martigues (0% versus 2.6% and 3%, respectively).

In order to explore the impact of various sensitizing aeroallergens in asthmatic children living in different environments, Ozkaya et al. conducted a cross-sectional study in Turkey [46]. The study involved patients aged 6–16 years old with a clinical diagnosis of mild-to-moderate asthma having required ICS treatment, residing in Istanbul, located on the coast, and in Erzurum, at an altitude of 1800–2000 m. Firstly, family and personal medical history were investigated through a questionnaire; then, all patients underwent skin prick testing for common aeroallergens (grass mixture including orchard grass, timothy grass, vernal grass, Kentucky blue grass, and rye grass; tree mixture incuding *Populus alba, Salix caprea, Olea europaea, Platanus vulgaris, Fraxinus excelsior, and Pinus silvestris*; cereal mixture including oat, wheat, barley, corn, and rye; weed mixture including mugwort, English plantain; mold; fungi mixture including *Alternaria, Aspergillus, Candida albicans*; cat and dog dander; cockroach, *Blattella germanica;* HDM *Dermatophagoides pteronyssinus*, *Dermatophagoides farinae*) and total serum IgE dosage. No significant difference between children living in Istanbul and those living in Erzurum was found in the prevalence of a family history of atopy, serum IgE levels, and asthma severity. On the contrary, the prevalence of atopy (defined as positive prick testing to at least one aeroallergen) was significantly higher in children living in Istanbul (80.6%) than in those living in Erzurum (49.6%) with an OR of 4.97 (IC 3.67–6.54). Within atopic subjects, the prevalence of sensitization to pollens was significantly higher in children living in Erzurum, whereas sensitization to HDM was significantly more frequent in children living in Istanbul.

Duenas-Meza et al. [47] analyzed data collected among children aged 6–15 years old, living at a high altitude (2500–3500 m) in Colombia, and suffering from severe asthma, which was defined according to International ERS/ATS guidelines [48]. The study protocol included a questionnaire that assessed respiratory clinical manifestations, the performance of baseline and post-bronchodilator spirometry, skin prick testing for common aeroallergens (*Aspergillus fumigatus, Dermatophagoides pteronyssinus, Dermatophagoides farinae, Blomia tropicalis, Lepidoglyphus destructor*, cat, horse and dog epithelium, *Cupressus arizonica*, *Ambrosia*, nut, egg, lentils, and wheat flour), total serum IgE dosage, and FENO measurements. Positivity to at least one prick test was found in a vast majority of patients (88.7%), and most of them (87.9%) were sensitized to at least one type of HDM. Obstruction in baseline spirometry was demonstrated in 31% of patients, despite the ongoing therapy for their severe asthma. These children had a significantly higher mean number of positive prick tests when compared to the group without obstruction. In addition, a weak but significant negative correlation between the number of positive SPT and the FEV_1_/FVC ratio was found (r = -0.34, 95% CI -0.55 - -0.09). Overall, this study showed a high prevalence of sensitization to HDM in asthmatic children living at high altitudes in Colombia. Even if taking into consideration patients suffering from severe asthma, this finding contrasts with those of the two studies previously discussed [45, 46]. The authors’ hypothesis is that tropical climatic conditions, such as higher temperature and humidity, could modify the effect of altitude on sensitization to HDM and on asthma when compared to children living at moderate altitudes in other countries, where the previous studies have been conducted.

An observational study has been published by Abiad et al. [49] evaluating sensitization to aeroallergens among the asthmatic pediatric population of Lebanon. Data collection took place between 2010 and 2017 and involved patients aged 1–18 years old with clinical (and if possible instrumental) diagnosis of asthma who underwent skin prick testing for a panel of 21 aeroallergens: 5 grasses (cocksfoot, sweet vernalgrass, rye-grass, meadow grass, timothy), *Parietaria*, olive, HDM (*Dermatophagoides pteronyssinu*s and *Dermatophagoides farinae)*, dog and cat danders, *Alternaria longipens*, *Aspergillus fumigatus* and *nidulans*, *Cupressaceae*, pine, German cockroach, and 4 cereals (oat, wheat, barley, maize). In this study, the prevalence of atopy was found to be very high (81.83% of patients with one or more positive prick tests), and the most frequent sensitizing allergen was HDM. When the study population was classified according to the living environment (< 900 m or > 900 m), children living at a lower altitude showed a statistically significant higher prevalence of sensitization to HDM, whereas those living at a higher altitude showed a statistically significant higher prevalence of sensitization to pollens. Moreover, the authors found a positive association between a patient’s age and the prevalence of atopy and of polysensitization, with non-atopic asthma being consequently more frequent in early childhood.

Ochoa-Avilés C. et al. [50] conducted a cross-sectional study in the Andean city of Cuenca (Ecuador), located at 2550 m above sea level, assessing the prevalence of asthma clinical manifestations through the ISAAC questionnaire and also performing skin prick testing: grass mix (*Dactylis glomerata, Festuca pratensis, Phoa pratensis, Phelum pratense, Lolium perenne*), tree mix (ash and salix), weed mix (*Plantago, Chenopodium, Artermisa, Ambrosia, Parietaria*), fungi (*Alternaria, Penicillum, Cladosporum*), dust mites (*Dermatophagoides pteronyssinus* and *Dermatophagoides farinae*), tropical mite (*Blomia tropicalis*), dog dander, cat, feather mix (chicken, duck, and goose), cockroach, and latex. The study population was composed of preschool children (aged 3–6 years old), all living in Cuenca. The reported prevalence of asthma was 17.8% and that of atopy (defined as one or more positive prick tests) was 33.5%, but a weak association was found between the two. In fact, non-atopic asthma was more frequent (10%) than atopic asthma (7.8%). This finding was probably due to the low age of the study population. The most frequent sensitizing aeroallergen was HDM, raising the same considerations previously mentioned concerning the work by Duenas-Meza et al. [47]. A noteworthy limitation of this study is that it did not compare the patients with matched ones living at lower altitudes.

In conclusion, not all studies discussed in this section found concordant results regarding the prevalence of asthma and atopy or concerning sensitization to specific aeroallergens, when comparing children living at different altitudes. When specific sensitizations were analyzed separately, a positive association between living at higher altitudes and sensitization to pollens and between living at lower altitudes and sensitization to HDM were confirmed, even if results were not univocal. A hypothesis is that tropical climatic conditions, such as higher temperature and humidity, could modify the effect of altitude on sensitization to HDM and on asthma when compared to children living at moderate altitudes in other countries, where the previous studies have been conducted.

## Conclusions

Despite a selection of the articles, the data we extracted were heterogeneous and, therefore, difficult to compare. In particular, the studied populations were located at very different altitudes with consequent different exposures to, e.g., air pollution, aeroallergens, and other specific characteristics of the mountain environment.

Concerning the studies included in Sect. 1, investigating the prevalence of asthma assessing respiratory clinical manifestations and previous clinical diagnoses of asthma, the beneficial role of living at a high altitude on the prevalence of pediatric asthma was confirmed. This aspect appeared stronger in regions where beneficial factors (e.g., air pollution, HDM exposure, mean temperature, and outdoor humidity) are more robust, such as in the Alps.

Moreover, the studies included in Sect. 2, including instrumental objective parameters concerning asthma, could confirm a good profile on airway inflammation in asthmatic children. This association is probably related to different factors coexisting at high altitudes, such as lower temperatures, humidity, and exposure to air pollution and allergens.

In contrast, according to the study analyzed in Sect. 3, evaluating the risk of hospitalization from asthma, a possible increase in hospitalization risk for asthma in children living at higher altitudes has been highlighted, although difficulties in obtaining timely primary and urgent care could have played a role.

Finally, the studies included in Sect. 4, assessing the prevalence of sensitization to specific aeroallergens, globally confirmed the positive association between higher altitude and sensitization to pollens and that between lower altitude and sensitization to HDM in pediatric patients, even if the results are not homogeneous, probably due to the different geographical and climatic regions considered.

At this point, the role of the climate change must be underlined: e.g., rising temperatures, humidity, and pollution levels could modify the characteristics of mountain, with different and increasing exposures [51, 52]. Consequently, these changes could lead to a possible influence on the development and severity of pediatric asthma in the future.

Several other environmental and lifestyle factors, such as exposure to cigarette smoke, respiratory infections and an unhealthy diet, contribute to the development of pediatric asthma through mechanisms only partially understood, also involving oxidative stress and pro-inflammatory triggers. Their impact on pediatric asthma development and severity represents a matter of critical importance, which deserves an intensive research effort [53].

Hence, further studies, e.g., extensive and international works, need to be conducted to better understand the complex interplay between different environmental factors, such as altitude, and the pathogenesis of asthma and how its prevalence and characteristics could vary due to climate change.

## Data Availability

Not applicable.

## Abbreviations

ENT: ear-nose-throat
FEV-1: forced expiratory volume in one second
FVC: forced vital capacity
FeNO: fractional exhaled NO
GNI: gross national income
GNP: gross national product
HDM: house dust mites
IOS: impulse oscillometry
ICS: inhaled corticosteroids
SPT: skin prick test
